# Family Carer Involvement in Dementia Care Research: A Scoping Review and Expert Consultation

**DOI:** 10.1111/hex.70741

**Published:** 2026-06-30

**Authors:** Franziska Anushi Jagoda, Julian Hirt, Claudia Mueller, Margareta Halek

**Affiliations:** ^1^ School of Nursing Science, Faculty of Health Witten/Herdecke University Witten Germany; ^2^ Department of Health Eastern Switzerland University of Applied Sciences St.Gallen Switzerland; ^3^ Pragmatic Evidence Lab, Research Centre for Clinical Neuroimmunology and Neuroscience Basel University Hospital Basel and University of Basel Basel Switzerland; ^4^ Institute of Health and Nursing Science, Medical Faculty Martin Luther University Halle‐Wittenberg Halle Saale Germany; ^5^ JBI Centre for Evidence‐Based Healthcare Eastern Switzerland University of Adelaide Adelaide Australia; ^6^ Institute for Information Systems University of Siegen Siegen Germany

**Keywords:** dementia, family carer, involvement, participation, research, scoping review

## Abstract

**Background:**

Family carers of people with dementia face particularly stressful and time‐intensive care situations, yet their involvement in research is vital to understanding and addressing their specific needs. To inform involvement approaches, it is important to understand activities, roles, barriers and enablers associated with involving this group. This scoping review aimed to identify, map and summarise evidence on the involvement of family carers in dementia care research.

**Methods:**

We conducted a scoping review following JBI methodological guidance. Empirical studies involving family carers of people with dementia who actively contributed to research processes were included irrespective of study design. We systematically searched MEDLINE, CINAHL, Scopus and PsycInfo, complemented by backward citation searching, searches of relevant evidence syntheses and an expert survey. Two researchers independently screened a randomly selected 20% of titles/abstracts and full texts. Data items were organised into six categories and guided the charting process. Findings were synthesised descriptively and narratively. Consultation with four family carers was carried out to contextualise and enrich the results.

**Results:**

Forty‐eight studies published between 2002 and 2026 met inclusion criteria, most using qualitative designs. Research contexts included development of social or digital interventions, Patient and Public Involvement, implementation, exploration, evaluation and adaptation. Forty‐five studies reported involvement during the execution phase, six in the preparatory and nine in the translational phase. In 35 studies, family carers acted as co‐thinkers, in 10 as partners and in 1 as decision‐makers. Seven studies reported barriers and enablers, while 17 reported impacts of involvement on carers and on the respective projects.

**Conclusion:**

Dementia care research involving family carers as co‐researchers shows heterogeneous and often limited methodological reporting. Meaningful involvement may benefit from careful considerations of roles, potential barriers and enablers and expected impacts. More research is needed on strategies that enable meaningful involvement and on formalised methods for recording and reporting impact.

**Patient or Public Contribution:**

The impetus for this scoping review came from a group of family carers of people with dementia who had worked with us in a previous project and shared with us their lived experiences and difficulties. In collaboration with them, we raised the questions that are described in the scoping review. During the scoping review, family carers were also asked to give their views on the findings and share their thoughts.

## 1 Background

Family carers of people with dementia provide most of the substantial and sustained support at home [1]. They advocate for people with dementia in all areas of daily life arising from high cognitive and functional dependency [2] and provide both physical and psychological support [3]. They also navigate the health care system to ensure personalised and appropriate care at home [4], for example, by assisting with medication, scheduling appointments and communicating with healthcare providers [1]. As a result, family carers have invaluable insights into the lived experiences and challenges of home‐based dementia care. Although caring can be rewarding [5], it also involves several challenges: Carers often find it difficult to leave their homes and maintain social networks due to their caregiving responsibilities [6]. They spend long hours providing care each day [7], which limits leisure time and contributes to social isolation [6]. Family carers frequently feel emotionally burdened [8], stressed [3], experience poor health [9] and show symptoms of depression and anxiety [1]. Together, these factors lead to a reduced quality of life for family carers [10].

Existing support services in the health care system do not seem to address this issue: Family carers still struggle to navigate the care system [6], and they perceive available care options as insufficient in quality and flexibility, making them too restrictive for the home care situation [11]. Research also suggests that the priorities of family carers have been overlooked in the development of supportive services [12].

One approach to improving this situation and focusing more directly on family carers and people with dementia is active involvement in research. Its importance has gained increasing recognition in health and social care, and many funding bodies in Western countries now require the involvement of patients in research [13]. Accordingly, interest in these approaches in dementia care research has also grown [14]. Such approaches help align individual preferences with collective decisions [15]. INVOLVE describes *public involvement* as ‘[…] research conducted “with” or “by” members of the public rather than “to,” “about” or “for” them’ [16]. The role of participants (co‐researchers) extends beyond that of a research subject. Interaction between researchers and co‐researchers is based on partnership, with research collaboratively designed. This approach, emphasising collaboration with the public, is increasingly seen as a democratic way to generate meaningful, contextually relevant knowledge, aligned with everyday life and significant outcomes for patients and carers [17]. Involvement in research allows laypeople to contribute across all stages of the process, from identifying research questions to disseminating findings, with involvement ranging from advisory roles to independent research tasks [18].

However, co‐researchers and academics report a lack of awareness regarding the purpose, roles and objectives of research involvement with people with dementia and their family carers [19]. Given the high time and cognitive demands on co‐researchers, the question arises of how family carers can be involved in research for their own benefit while actively shaping it. Considering their limited resources, responsibilities and individual challenges, discussing best practices for involvement is essential: roles, tasks and responsibilities need clarification, and barriers must be identified [20]. Several reviews have examined involvement approaches in dementia research from various angles. Miah et al. [21] mapped patient and public involvement in European dementia research, while Groothuijse et al. [22] focused on methods for active involvement of people with dementia and long‐term care users. Bethell et al. [23] reviewed research methods more broadly. Despite the growing body of literature, these reviews have not centred on family carers providing home‐based care for people with dementia as co‐researchers, a gap that the present scoping review aims to fill.

To our knowledge, this is the first scoping review addressing the following research question: What is known about the extent, range and nature of research activity in the area of home‐based dementia care research involving family carers as co‐researchers?

The three sub‐questions are as follows:

1. What strategies have been used by the research team to involve family carers of people with dementia in research?
2. What roles, barriers and enablers are being reported to involve family carers of people with dementia in research?
3. What impact can researchers and family carers of people with dementia describe as a result of the research involvement?

## 2 Methods

### 2.1 Design

To identify, map and summarise the evidence on the involvement of family carers in dementia care research, we chose a scoping review design guided by the JBI methodology [24]. We structured our report according to the Preferred Reporting Items for Systematic Reviews and Meta‐Analyses extension for Scoping Reviews [25].

We developed a protocol for this scoping review [26] and registered it on the Open Science Framework: https://doi.org/10.17605/OSF.IO/PMZYV.

### 2.2 Eligibility Criteria

Eligibility criteria can be viewed in Table 1. Of the studies with mixed samples (family carers and people with dementia), we only included those where we could judge that family carers were in the majority (i.e., > 50% of all participants). With regard to involvement in research, studies were included regardless of whether they reported a formal framework or definition of involvement, in line with scoping review methodology aimed at mapping the breadth and range of evidence.

**Table 1:** Exclusion and inclusion criteria.

Domain	Inclusion	Exclusion
Participants *Study population*	Family carers of people with dementia (e.g., partners, children, friends, neighbours)	family carers of people with conditions other than dementia; dyadic involvement
	All forms and severities of dementia	
**C**oncept *Intervention*	Research involvement as an active research partner throughout the research cycle (preparatory phase, execution phase and translational phase)	Research involvement as a research subject
**C**ontext *Setting*	Research in the context of providing care for a person with dementia	Research not related to dementia care, i.e., research related to dementia, biomedical and genetic research, prevalence, epidemiology
	Community home setting	Institutional care setting (e.g., nursing or care home, day care)
Sources of Evidence	Empirical study design (i.e., qualitative, quantitative and mixed methods design; irrespective of study size)	Evidence synthesis
	Original research as indicated by an introduction, methods, results and discussion (IMRaD) structure, including study protocols, corrigenda and errata	Opinion pieces (e.g., commentary, editorial)
	Journal articles	Other publication types and grey literature (e.g., thesis, book, internet report)
	German and English languages	Other languages
	No restriction on publication year	
	No restriction on the country of study conduct	

### 2.3 Information Sources and Search Strategy

We searched MEDLINE (via PubMed), CINAHL, Scopus and PsycINFO (via EBSCO) without filters (last search: 11 March 2026). The search strategy, developed by F.J. and J.H., both experts in the field, covered three concepts: family carers, dementia and involvement, with synonyms identified through an initial MEDLINE scan. We also conducted one round of backward citation searching in Scopus based on all included studies and pertinent evidence syntheses [27] (last search: 4 May 2026). Full database search strategies are provided in Supporting Information S4: Appendix. In addition, we contacted 53 experts in involvement in research, identified through key organisations (Canadian Institute of Health Research, Canada; Patient‐Centred Outcomes Research Institute, USA; PartNet, Germany; National Institute for Health Research, UK; The Dementias and Neurodegenerative Diseases Research Network, UK; Research Centre for Patient Involvement, Denmark; European Patients’ Forum, EU; INTERDEM, EU; International Collaboration for Participatory Health Research) and eligible study authors to suggest further relevant studies (December 2023; following our initial and database and supplementary search).

### 2.4 Selection of Sources of Evidence

All identified references were collected and stored in EndNote X9 (Clarivate Analytics, PA, USA), and duplicates were removed. We screened the studies using the Rayyan web application (Rayyan) [28]. A randomly selected 20% of titles/abstracts and full texts were independently screened for eligibility by two reviewers (F.J. and J.H.) to ensure consistency in the application of the predefined inclusion and exclusion criteria. Discrepancies were resolved through discussion and consensus, involving a third reviewer (M.H) where necessary. The remaining references were screened by one reviewer (F.J.). Any uncertainties arising during single‐reviewer screening were discussed within the research team. No systematic disagreements were identified during the double‐screening process. This approach is consistent with JBI methodological guidance for scoping reviews, which allows partial double‐screening to ensure the reliability of study selection [24].

### 2.5 Data Charting Process and Data Items

The research team jointly developed a data extraction sheet informed by three sources: the JBI Manual for Evidence Synthesis [29], the methodological guidance by Pollock et al. [30] and the GRIPP2 reporting checklist [31]. The framework of Shippee et al. [32] on user engagement phases (preparatory, execution, dissemination/translational) structured the extraction process. One reviewer (F.J.) extracted all quotations, and 10% of included studies were double‐checked by a second reviewer (J.H.); discrepancies were resolved by consensus.

Data items were grouped into six categories: (1) citation details (author, journal, year, location, family carer co‐authorship); (2) study characteristics (aim, design); (3) involvement characteristics (sample, strategies for involvement, research phase, framework/definition used, activities, frequency, roles [20], compensation); (4) family carers’ reflections (method, barriers, enablers, overall perceptions); (5) researchers’ reflections on involvement (same subcategories); and (6) impacts of involvement (definitions, impacts reported by researchers and carers and methods used to collect impact data). Descriptive citation details, study characteristics, involvement characteristics (sample, strategies, framework/definition used, compensation) and methods to collect data on reflections and impacts were directly extracted from the included articles. More in‐depth information on barriers, enablers and impact was also extracted as reported in the studies and subsequently grouped into thematic categories to enable comparison across studies. In contrast, aspects such as involvement phases and roles of family carers were classified by the review team based on predefined frameworks (see Section 2.6). The full data charting plan is provided in Supporting Information S2: Appendix.

### 2.6 Synthesis of Results

**Figure 1:**
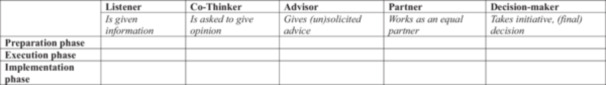
Involvement matrix based on Smits et al. (2020); own visualisation.

To provide an overview of the included studies and a detailed analysis of involvement activities, phases, roles and barriers/enablers, results were synthesised as follows. Citation and study characteristics were summarised descriptively using tables and figures. We also produced a descriptive summary of all 48 papers, including geographical origin, year of publication, family carer co‐authorship, study design, target group and study topic. Characteristics of involvement were analysed descriptively and narratively. For the narrative analysis of roles, phases and activities, we applied the Involvement Matrix by Smits et al. [20], which outlines five roles and three phases (Figure 1). Study information was mapped to the corresponding matrix elements. The role of *listener* did not apply since our definition of research involvement excluded this role. Reflections on involvement (from carers and researchers) and impacts on carers and research projects were analysed thematically and are presented narratively.

### 2.7 Consultation With Family Carers of People With Dementia

In line with the aim of this scoping review, four family carers of people with dementia were consulted as part of the study design and interpretation of results. This consultation was designed as an exploratory phase to contextualise and reflect on the scoping review findings rather than to achieve generalised findings. Although the literature search was updated, the additional studies did not substantially change the overall patterns and themes identified in the review. Therefore, a second consultation round was not conducted. The consultation was conducted via video call using Zoom, moderated by the lead researcher (F.J.), using the platform's whiteboard to visualise key points. Five themes were discussed: strategies, roles, barriers, enablers and impact. Carers rated the relevance of each theme on a three‐point scale (*not particularly important*, *important* and *very important*) in accordance with Harding et al. [33], followed by an open discussion. The consultation proceeded in two steps: the carers’ initial assessment and rationale, and subsequent reflection after being informed about how frequently each theme was addressed in the included studies. Sessions were audio‐recorded, transcribed automatically (F4) and analysed using qualitative content analysis [34] by F.J. using MAXQDA. Carers’ feedback informed the interpretation and discussion of results, but they were not involved in screening or data extraction. The involvement of family carers is reported in accordance with the GRIPP 2 short form checklist [31] to ensure transparency and systematic reporting.

### 2.8 Deviations From the Protocol

Alterations to the protocol can be found in Supporting Information S3: Appendix; the main changes relate to additions to the data analysis and the narrative analysis of the included papers.

## 3 Results

### 3.1 Selection of Sources of Evidence

**Figure 2:**
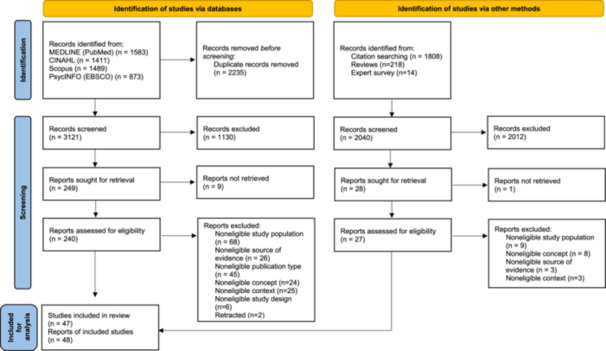
PRISMA flow diagram.

The database searches yielded 5356 records. After removing duplicates, we screened 3121 titles and abstracts, assessed 84 full texts and included 43 records. Additional sources yielded 2040 records; 27 full texts were assessed, and 5 were included. In total, 48 references reporting on 47 studies were incorporated into the narrative synthesis (Figure 2: PRISMA flowchart of study selection).

### 3.2 Characteristics of Sources of Evidence

Study characteristics are shown in Table 2. Most studies were conducted in the UK (*n* = 21; 44%), followed by Australia (*n* = 9; 19%) and Canada (*n* = 4; 8%). Other studies were from the USA (*n* = 3), the Netherlands, Sweden and Europe (*n* = 2 each), and Portugal, Estonia, Ireland, Germany and India (*n* = 1 each). The median year of publication was 2020 (range: 2009–2026).

**Table 2:** Characteristic of the studies included in the scoping review.

Authors	Year	Study design	Study location	Study aim and focus	Who was engaged?	Carer sample
Arcia, A. et al.	2019	qualitative design; participatory design	USA	development of pictograms; focus on family carers	family carers	16
Akter, S. et al.	2025	qualitative design; co‐design	Australia	inform core elements of the project's model of care and its implementation and evaluation; focus on family carers and person with dementia	family carers	10
Bala, N. et al.	2025	qualitative design; community‐based participatory research, human‐centred design	Australia	development of a dementia care and support needs framework; focus on family carers and people with dementia	family carers with persons with dementia	27
Banbury, A. et al.	2021	qualitative design; co‐design	Australia	development of a peer support programme; focus on family carers	family carers	6
Baruah, U. et al.	2021	qualitative design;	India	adaption of a training and support intervention for family carers; focus on family carers	family carers	28
Bilodeau, G. et al.	2019	mixed‐methods design	Canada	development of decision boxes; focus on family carer and person with dementia	family carers as a dyad with persons with dementia	27
Burnell, K. J. et al.	2015	qualitative design	UK	development of a peer support intervention; focus on family carers	current and former family carers; members of voluntary organisations, clinical health professionals and an academic	12
Cations, M. et al.	2018	mixed‐methods design	Australia	implement and sustain improvements in post‐diagnosis care; focus on family carers and person with dementia	family carers with persons with dementia where possible; or just the family carer	protocol
Dale, J. et al.	2018	qualitative design; co‐production	UK	development of a web‐based programme for carers; focus on family carers	current or former family carers	60
Dalgarno, E. L. et al.	2021	qualitative design	UK	examination of formal home care; focus on family carers	current or former family carers	5
Davies, K. et al.	2021	qualitative design	UK	evaluate the usability, usefulness and relevance of HMN; focus on people living with early‐stage dementia	family carers with persons with dementia	protocol
Davies, N. et al.	2016	qualitative design; iterative co‐production	UK	development of a prototype website towards the end of life; focus on family carers	current and former family carers	11
Davies, N. et al.	2019	qualitative design; iterative co‐design	UK	development of a toolkit regarding decision‐making at the end of life; focus on people with dementia	family carers	4+ undefined number
Davies, N. et al.	2021	qualitative design; coproduction approach	UK	development of a decision aid for family carers towards end‐of‐life; focus on family carers	family carers in an experts by experience group	4
Davies, N. et al.	2024	qualitative design; co‐design approach	UK	description of co‐design processes to construct a care‐framework delivered through. a digital app; focus on family carers and people with dementia	family carers with people with dementia in a PPI group	7
Di Lorito, C. et al.	2020	qualitative design; co‐research	UK	proposition of a model for good practice in co‐research; focus on family carers	people with lived experience of caring for someone with dementia	2
Giebel, C. et al.	2019	qualitative design	UK	dissemination of lessons of two PPI groups: focus on family carers and people with dementia	current and former family carers in a group with persons with dementia	20
Goh, A. M. et al.	2022	qualitative design; co‐Design	Australia	description of a methodology used in the co‐design of a dementia training package; focus on home care workers	family carers	34
Griffiths, Sarah et al.	2022	qualitative design	UK	critical appraisal of the realist methods they used; focus on people with dementia	current and former family carers and dyads	17
Guerra, S. R. et al.	2013	qualitative design	Portugal	evaluation of the clinical relevance and benefits of a psychoeducational programme; focus on family carers	family carers	6
Hales, S. A. and Fossey, J.	2018	qualitative design	UK	development of the Caring for Me and You package; focus on family carers	family carers	29
Harding, A. J. E. et al.	2018	qualitative design; co‐Design	UK	scope needs and perspectives in an envisaged future with AAL support; focus on family carers	family carers	6
Hwang, A. S. et al.	2012	qualitative design; participatory design	Canada	discussion of methods and presentation of results as tensions; focus on family carers	family carers	6
Hwang, A. S. et al.	2015	mixed‐methods design	Canada	development of a core outcome set; focus on people with dementia	family carers; if their partner with dementia had been recruited to the study	protocol
Kelly, S. et al.	2015	no clear design	UK	identification of a top 10 prioritised list of uncertainties, priority setting in research; focus: dementia care	family carer	1188
Keogh, F. et al.	2021	no clear design	Ireland	create a pathway for the voice and experience of PWD and FC; focus on family carers and people with dementia	family carers	28
Kerkhof, Y. et al.	2019	qualitative design; participatory design	The Netherlands	development of an interactive selection tool (web application); focus on people with dementia	family carers with persons with dementia	8
Kort, Helianthe S. M. and van Hoof, Joost	2014	no clear design	The Netherlands	description of the creation of a website to offer information on home modifications; focus family carers and people with dementia	family carers	32
Kowe, A. et al.	2021	qualitative design; design approach	Germany	description of a participatory data analysis; focus on family carers	family carers	6
Lindauer, A. et al.	2023	no clear design	USA	revision of an intervention to fit FTD care partners; focus on family carers	family carers	15
Liddle, J. et al.	2022	qualitative design; participatory design	Australia	exploration of current experiences and contexts of use of technologies; focus on family carers and people with dementia	family carers in a reference group	unclear
Lord, K. et al.	2022	mixed‐methods design	UK	exploration of the experiences of people affected by dementia; focus on family carers and people with dementia	current or former family carers	15
Miah, J. et al.	2018	qualitative design	Europe	identification of PPI impact; focus dementia research	family carers and persons with dementia	12
Miah, J. et al.	2020	qualitative design	Europe	evaluation of the acceptability and perceived outcomes of Research Awareness Training; focus on people with dementia	family carers and persons with dementia	protocol
Molinari‐Ulate, M. et al.	2022	qualitative design	UK	exploration of e‐PPI within a dementia‐specific context; focus on dementia care	family carers	11
Mountain, Gail A. & Craig, Claire L.	2012	qualitative design	UK	working collaboratively with people with dementia to obtain insights for informing potential intervention topics; focusing on people with dementia	family carers and persons with dementia	8
Murfield, J. et al.	2022	qualitative design	Australia	planning and designing an intervention in the early stage; focus on family carer	family carers	6
Parveen, S. et al.	2018	no clear design	UK	reporting the process of involving a diverse range of experts, focusing on dementia care	family carers as part of a group with support workers, people with dementia etc	6
Pol et al.	2014	qualitative design	UK	description of a PPI process; focus family carer	current or former family carers	9
Rapaport, P. et al.	2018	qualitative design	UK	reflection of a co‐production process; focus on people with dementia	current and former family carers	9
Rathnayake, S. et al.	2021	mixed‐methods design	Australia	reporting on the co‐design of an mHealth application; focus family carer	family carers	176
Robinson, L. et al.	2009	qualitative design; participatory design	UK	creation of an acceptable and effective prototype; focus on people with dementia	family carers with persons with dementia	11
Svedin, F. et al.	2021	mixed‐methods design	Sweden	adaption of a British intervention for the Swedish context; focus on family carers and people with dementia	family carers	10
Svedin, F. et al.	2025	mixed‐methods design	Sweden	exploration of the experience, process and impacts of involving carers as public contributors during intervention development phase; focus on family carers	family carers	4
Thodis, A. et al.	2026	mixed‐methods design	Australia	assessment of website's usability, accessibility and ease of navigation; focus family carers	family carers	30
Varik, M. et al.	2021	qualitative design	Estonia	reflection on experiences from the participatory action research; focus on family carers	family carers	16
Webkamigad, S. et al.	2020	qualitative design	Canada	development of health promotion materials about dementia for Indigenous peoples; focus on dementia care	family carers	5
White, C. L. et al.	2018	no clear design	USA	description of public engagement for an implementation plan; focus family carer	family carers	28

Ten studies (21%) listed family carers as co‐authors: eight as individuals and two as groups, for example, from self‐help organisations.

Regarding study design, most studies were qualitative (*n* = 34; 71%), followed by mixed‐methods (*n* = 8; 17%). Six studies did not report a clear design. Among qualitative studies, additional details included participatory design, (iterative) co‐design, (iterative) co‐production, co‐research, community‐based participatory action research, human‐centred design and design approaches.

Regarding the study aim, most studies (*n* = 21; 44%) focused solely on family carers. The remaining studies addressed the person with dementia (*n* = 9; 18%), both carer and person with dementia (*n* = 10; 21%), dementia care in general (*n* = 4; 8%), or family carers and dementia research in general (*n* = 2 each). In terms of topic, half (*n* = 26; 54%) examined social and/or digital intervention development, a third (*n* = 14; 29%) focused on PPI research and the remaining eight studies addressed implementation, exploration, identification, or adoption.

### 3.3 Involvement Characteristics

In addition to family carers being the main group involved, some studies included both carers and people with dementia (*n* = 10; 20%). In a few studies (*n* = 3; 6%), carers participated as part of a group with members of voluntary organisations [35], clinical health professionals [35] or support workers [36].

Regarding theoretical background, more than half of the studies (*n* = 29; 60%) reported a framework or definition of involvement. The most frequently cited were Co‐Design (*n* = 7; 15%), INVOLVE (*n* = 5; 10%), Participatory Action Research (*n* = 5; 10%), Co‐Production (*n* = 4; 8%) and PPI (*n* = 2; 4%). Other frameworks were mentioned once each, such as User‐Centred Design [37], Participatory Methods [38], the model of Cornwell and Jewkes (1995) [39], Participatory Design [40], Public Engagement [41] and Guidance from the National Institute for Health and Care Excellence [42]. Nearly half of the studies (*n* = 19; 40%) reported no framework or definition of involvement.

Regarding training for family carers as lay researchers, 3 of the 48 studies (6%) provided training. This included either unspecified sessions on research processes and methods [43, 44] or six 1‐h sessions on key research concepts [45].

Regarding the frequency of involvement, most studies (*n* = 38; 79%) engaged family carers multiple times or throughout the research process, while in 10 studies (21%), carers were involved only once.

Regarding compensation, most studies (*n* = 36; 75%) did not report any reimbursement for family carers. In 12 studies, carers received compensation such as money (*n* = 9) and/or travel expenses (*n* = 5). Three studies reported compensation but did not specify the type.

Regarding strategies to engage family carers, six studies (13%) reported specific approaches, including virtual meetings [46, 47], individual meetings [48], training [43], safe spaces for co‐researchers [43] and developing a common understanding [49]. The authors did not provide any evaluations or reflections on these strategies.

Regarding the research cycle phase, 45 studies (94%) described family carers’ involvement in the execution phase of the study; 6 studies (15%) described involvement in the preparatory phase, and 9 studies in the translational/dissemination phase (19%). Studies involving carers in the preparatory or translational phases generally also included them throughout the rest of the study.

Regarding the roles adopted by family carers, based on the framework by Smits et al. [20], family carers often adopted multiple roles within the studies: co‐thinker in 35 studies (73%), advisor in 24 studies (50%), partner in 10 studies (21%) and decision‐maker in 1 study (2%).

Table 3 presents the involvement matrix [20], which we expanded to include the specific activities through which family carers contributed. Carers acted as co‐thinkers in focus groups, interviews, surveys and workshops; as advisors, providing feedback on study design, methods and data analysis; as partners, actively contributing to study preparation, execution and dissemination; and as decision‐makers, presenting study results during the translational phase.

**Table 3:** Roles and research project phase.

	Preparation phase	Execution phase	Activity	Translational phase
CO‐THINKER		Focus groups (Akter et al., 2025; Davies et al., 2021; Davies et al., 2016; Griffiths et al., 2022; Guerra et al., 2012; Hales et al., 2018; Harding et al., 2018; Kort et al., 2014; Miah et al., 2018; Rapaport et al., 2018; Rathnayake et al., 2021; Robinson et al., 2009; Webkamigad et al., 2020; White et al., 2018)	‒ reflecting (Guerra et al., 2012), ‒ consensus building (Harding et al., 2018), ‒ testing interventions (Hales et al., 2018), ‒ usability/impressions (Kort et al., 2014), ‒ content rating (Rathnayake et al., 2021)	
		Discussion groups (Burnell et al., 2015; Dale et al., 2018; Guerra et al., 2012; Keogh et al., 2021; Liddle et al., 2022; Lord et al., 2022; Mountain et al., 2012; Parveen et al., 2018)	‒ providing feedback on methods and engagement in data analysis (Liddle et al., 2022) ‒ topic guide reflection (Liddle et al., 2022) ‒ topic guide was piloted with carers (Lord et al., 2022)	
		Think‐aloud approaches (Davies et al., 2016; Davies et al., 2021; Kerkhof et al., 2019)		
		Semi‐structured interviews in person or by telephone (Banbury et al., 2021; Cations et al., 2018; Griffiths et al., 2022; Rathnayake et al., 2021; Svedin et al., 2021; Varik et al., 2021; Webkamigad et al., 2020)		
		Survey (Burnell et al., 2015; Harding et al., 2018; Kelly et al., 2015; Miah et al., 2020; Rathnayake et al., 2021; Varik et al., 2021; White et al., 2018)	‒ providing input on a draft of a coding framework (Miah et al., 2020)	
		(Design) Workshops [requirements, usability, intervention development] (Bilodeau et al., 2019; Cations et al., 2018; Dale et al., 2018; Hwang et al., 2015; Hwang et al., 2012; Molinari‐Ulate et al., 2022; Pol et al., 2014)		
		‒ study promotion (Parveen et al., 2018)		
ADVISOR		Designing the study		‒ input on the publication of the paper; suggested the use of diagrams to illustrate main findings (Miah et al., 2020)
		‒ Critical feedback for study design (Bilodeau et al., 2019) ‒ coinvestigator who participated in the study design (Bilodeau et al., 2019) ‒ Voting on research topics (Keogh et al., 2021) ‒ set focus for evidence synthesis (Davies et al., 2019)		
		Data collection/development		
		‒ topic guide development (Pol et al., 2014) ‒ Co‐Designing/Prototype designing (Arcia et al., 2019; Bala, et al., 2025; Banbury et al., 2021; Baruah et al., 2021; Davies et al., 2016; Davies et al., 2021; Goh et al., 2022; Murfield et al., 2022; Thodis et al., 2026) ‒ discussion groups with likert‐type ratings (Burnell et al., 2015) ‒ advising and testing data collection methods (Dalgarno et al., 2021; Davies et al., 2019) ‒ development of caregiver scenarios and personas (Murfield et al., 2022) ‒ designing and finalising content for information material (Parveen et al., 2018) ‒ scenario work; artefact analysis (Robinson et al., 2009)		
		Data analysis		
		‒ think tank to help interpret data (Davies et al., 2019)		
		Ongoing		
		‒ Advisory committee (Cations et al., 2018; Dale et al., 2018; Davies et al., 2024; Giebel et al., 2017; Goh et al., 2022; Svedin et al., 2021; Varik et al., 2021)		
PARTNER	‒ Protocol preparation and feedback on proposal (DiLorito et al., 2020; Giebel et al., 2017)	‒ Co‐interviewing (DiLorito et al., 2020) ‒ Actively involved in data analysis (DiLorito et al., 2020; Kowe et al., 2021; Lord et al., 2022) ‒ Developing questions from a discussion for a survey (Dalgarno et al., 2021) ‒ Reviewing an information programme and making amendments (Banbury et al., 2021)		‒ Dissemination work (Davies et al., 2021) – not specified ‒ co‐author of a paper (DiLorito et al., 2020; Giebel et al., 2017; Liddle et al., 2022; Lord et al., 2022) ‒ supporting during the implementation stage (Cations et al., 2018)
DECISION‐MAKER				‒ presenting at conference (Parveen et al., 2018)

### 3.4 Family Carers’ and Researchers’ Reflection on Involvement

#### 3.4.1 Family Carers’ Reflection

Nineteen per cent of studies (*n* = 9) reported family carers' reflection on their research involvement. Of these, four reported the methods used: evaluation forms [50], the Public and Patient Engagement Evaluation Tool [51] and interviews [21, 51, 52].

Family carers reported barriers to involvement in seven studies (15%), including personal, methodical and communication aspects. Personal barriers included time away from home [43], lack of self‐confidence [36], difficulties with technology and dislike of virtual meetings [53]. Methodical barriers included methods that were difficult to understand [54], overlapping themes and unclear questions in workshops [50], a missing sense of group in digital settings [52, 53], and researchers not being open to input from the public, leading family carers to feel predetermined outcomes [51]. Communication barriers included long silent gaps between meetings and insufficient clarity about project progress [51] and contribution [52].

Family carers reported enablers in seven studies (15%): Enablers included methodological, personal and contextual factors. Methodological enablers cited by family carers included telehealth to involve participants remotely [52, 46], preparation and training for lay researchers [43, 54], debriefing after workshops [43], sufficient time to exchange ideas and issues [50], advance outlines of workshop content [50], use of everyday language [54] and seeing that their contributions impacted the research [21, 51]. Personal enablers included researchers building confidence in lay researchers [43] and providing reassurance, which helped family carers feel comfortable, valued and able to express their views [51]. Contextual enablers included group diversity [46], a down‐to‐earth workshop atmosphere, emphasising the process as a learning curve, a visual project timeline and peer support through group work, a visual timeline of the progress of the whole research project, and group work as peer support [51].

Three studies reported family carers’ overall perceptions of involvement. Family carers described the experience as positive, noting connections within their diverse groups [46], satisfaction and feeling able to contribute as co‐designers [50]. In one study, family carers highlighted a lack of group diversity and wished for more information on the impact of their contributions [51].

#### 3.4.2 Researchers’ Reflection on Family Carers’ Involvement

Ten per cent of studies (*n* = 5) reported researchers’ reflections on family carers’ involvement. Two studies described the methods used: observations and discussions [50] and semi‐structured interviews [52].

Researchers reported barriers to family carers’ involvement in four studies, including the need for more information about the co‐design process [46], time constraints due to ongoing caregiving limiting travel to meetings [35, 48] and the inability to process all ideas in limited research projects [50].

In four studies, researchers reported enablers of family carers’ involvement, including a pragmatic approach to co‐production, for example, individual meetings to reduce burden [48], time for group‐building [46], a research team attentive to each other's and carers’ views [50], an experienced family carer chair as partner [50] and providing frequent feedback [52].

One study reported researchers’ overall perception of family carers’ involvement, noting that it was challenging to include multiple stakeholder groups as co‐design partners [50].

### 3.5 Impacts of Involvement

Thirty‐five per cent of studies (*n* = 17) reported on the impacts of family carers’ involvement, either from researchers’ or family carers’ perspectives. None of these studies defined *impact*.

#### 3.5.1 Perspective of the Researchers

Of the 17 studies, 16 studies reported on the impact described from the researchers’ point of view; two studies reported on the formal measurement of these statements: in both studies, researchers were interviewed [52, 55]. The impact that the family carers’ involvement had was a change of an intervention name [35], identification of important issues in care that were not considered by the researchers beforehand [35, 44, 56] and providing researchers with the perspective of living with dementia [35, 44, 52, 56], proposition of practical ways and making the intervention as appropriate and feasible as possible to the target recipients [35, 57]. Other aspects mentioned were creating acceptable and appropriate content for an intervention [48], increasing the rigour in the development process [49], making research documents and topic guides more language‐appropriate for participants, and contributing to diffuse tension in interviews, because family carers seemed at ease opening up with someone experiencing a similar journey [43]. The family carers’ involvement enabled researchers to get a deeper understanding of the participants [38], affirmed the personhood of the participants and embedded the message of equality and shared power in the research project. It also led to the use of non‐traditional recording and dissemination methods, such as film or illustrations [58] and to the refinement of the content and wording of public information [56]. Further impacts mentioned by the researchers were an improvement in the depth of data quality by co‐researchers adding additional results during data analysis [54], technology could be developed to be easy and attractive in use [59], the involvement provided insight into what participants think of the researchers work [56] and it led to researchers changing future practices, such as taking people's perspectives into account in planning and prioritising research [56]. Additional impact mentioned was a success in recruitment, not only with regard to the sample size but also to the sample diversity, which led to richer qualitative data [36, 44], members expressing a desire to continue being involved with research once the study was completed [36], and the involvement group being a social network outside of meetings [60].

#### 3.5.2 Perspective of Family Carers

Of the 17 studies, 7 studies reported on the impacts of the involvement as described by family carers themselves; 2 studies reported on the measurement using interviews with family carers to describe the impact [46, 52]. The family carers’ involvement challenged the participants’ group's perception of their circumstances and reciprocally helped them validate their feelings [46] since they were able to explore, interpret and communicate their feelings [38]. The involvement made family carers feel fulfilled, motivated and worthy, as they could give back to the community [43, 52, 56]. The empathy that was created between family carers enabled the group to relax and share relevant thoughts with each other; family carers also gained confidence throughout this involvement process [43]. This process also encouraged them to adopt a more active role in representing themselves, and family carers found a new way to dialogue about dementia [38]. Family carers also felt heard, valued and like they had a voice [58]. They gained knowledge [52, 56], were able to influence intervention manual content [51] and made written materials more accessible for participants [56]. Lastly, they felt they provided researchers with a better understanding of the impact of dementia [56].

Across perspectives, both researchers and family carers noted that involvement enhanced research relevance and quality and provided meaningful personal experiences. Researchers focused on practical and methodological outcomes, whereas family carers emphasised emotional, social and self‐efficacy outcomes. Divergence occurs in emphasis: researchers highlight study design, data quality and feasibility; family carers highlight personal growth, voice and social connection. Convergence is seen in areas such as understanding participant experiences, shaping interventions and contributing to meaningful research outcomes.

### 3.6 Consultation of Family Carers of People With Dementia

The consultation with four family carers of people with dementia took place on 9 and 11 December 2024. The detailed results can be found in the Supporting Information S1: Appendix. The consultation with family carers helped contextualise the findings of the scoping review and provided additional critical reflections on roles, barriers, enablers, strategies and perceived impact of involvement.

All four family carers felt that it was important for researchers to consider strategies for involving family carers as co‐researchers. It was important to them that involvement in research would not be done spontaneously or on instinct; otherwise, the strategies may not be well thought out. Researchers should consider and think about how to improve the accessibility of research projects for family carers. This perspective aligns with the scoping review finding, which showed that only 6 out of 48 references explicitly reported on strategies to engage family carers, highlighting a gap in structured approaches.

All four family carers felt that it was very important for researchers to consider the level of involvement and the different roles of family carers in a research project. For them, this would be a key aspect of involvement in research. Transparency in this respect would also be fair to the co‐researchers in research projects. Family carers were not surprised by the distribution of roles in the included studies. As the responsibility and effort involved in the roles increases, the number of family carers would understandably decrease, as this would also require time and cognitive effort. This reflects the scoping review findings, where co‐thinker and advisor roles were most common, while partner and decision‐maker roles were less frequent. Three of the four family carers felt that it was very important for researchers to address enablers to their involvement. They are heavily involved in acute care situations, which makes it difficult to be involved in research. Even if they wanted to, if the circumstances were not right, they would not be able to. The research context would always be secondary, so researchers should offer support to enable their role as family carers. In the worst‐case scenario, family carers would also feel tokenistically involved if their situation at home was not sufficiently considered. This complements the scoping review findings, which indicated that few studies explicitly reported enablers for involvement, despite their importance for meaningful involvement.

Three of the four family carers felt it was very important for researchers to consider barriers to involvement. One family carer noted that discussing barriers at the end of the project might be more relevant from the researcher's perspective than the family carer's, since the project would be complete for them. The family carers believed that addressing barriers was as important as considering enablers. This aligns with the review findings, which highlighted known barriers such as time constraints and caregiving responsibilities, yet few studies systematically addressed them.

In terms of impact, family carers felt that for the role of co‐thinker and advisor, the magnitude of impact was less important. They would share their thoughts regardless. For the roles of partner and decision‐maker, however, impact mattered more, as their personal investment was higher. Family carers also noted that researchers seemed to think it was important to report impact, but they would like clearer reporting to show whether their contributions had been adequately considered. This complements the scoping review results, which found that impact was rarely defined or formally measured and that researchers’ and carers’ perspectives often diverged.

## 4 Discussion

It should be acknowledged that many included studies provided limited or heterogeneous reporting of involvement methods and outcomes, which constrains the extent to which conceptual or methodological shortcomings can be firmly established. This limitation has also been noted elsewhere [21, 61]. Consequently, the findings of this scoping review should be interpreted as preliminary rather than definitive. The identified patterns provide a first overview of the current evidence base, while further conceptual and methodological research is needed to better understand involvement practices in dementia care research.

Within these limitations, the scoping review nevertheless identified several recurring patterns regarding roles, phases of involvement, barriers, enablers and impact. In the current research landscape, family carers of people with dementia are most frequently involved as co‐thinkers during the execution phase of research projects, with limited involvement in other roles or phases. These findings are consistent with those of other studies and reviews on involvement in dementia research [62, 63, 64, 65]. The relatively low involvement of family carers in the dissemination phase has also been described elsewhere [62, 63]. This may reflect the timing of research projects, as dissemination often occurs once the project has been completed, at which point family carers are usually no longer available. Nevertheless, providing opportunities for involvement according to family carers’ abilities and wishes could help achieve a more balanced involvement across project phases and roles.

Few of the included studies explicitly reported strategies specifically designed to involve family carers in research, and where mentioned, strategies were often minimally described or poorly theoretically framed. This aligns with the consultation, in which family carers emphasised the need for thoughtful, well‐planned strategies to support their involvement. Family carers of people with dementia play a key role in providing home care and therefore have many responsibilities [1], and involvement as co‐researchers represents an additional time and cognitive burden [66, 67]. A lack of structured strategies or considerations of barriers and enablers can reduce the effectiveness of involvement and may limit family carers’ involvement. Conversely, adaptive approaches that consider flexibility in scheduling and roles and foster meaningful relationships within research teams appear to support more effective involvement [63, 67, 68, 69]. Training, authentic partnerships, trust and use of technology were highlighted as key enablers, while time constraints, lack of self‐confidence and complex language were noted as common barriers [63, 65, 69, 70]. It should be noted, however, that these aspects were often reported as incidental observations rather than systematically collected data.

Barriers and enablers of involvement in research with family carers of people with dementia were rarely the primary focus of the included studies, contrasting with the consultation findings in which family carers highlighted the importance of researcher support as recognition of their contributions. Similar observations have been made in other contexts, such as stroke research, where only a minority of studies reported barriers and enablers for research involvement [65]. Process evaluations in future research should aim to systematically capture these factors to optimise family carers’ involvement. Similar aspects to those identified as barriers and enablers in this scoping review can also be found in the literature in connection with other groups that were involved as co‐researchers. Barriers include time constraints [65], lack of self‐confidence [68] and technical language and jargon [67] and enablers such as training [65], building an authentic partnership and trust [65] and using technology [63] seem to be important.

Regarding impact, fewer than half of the studies reported on the outcomes of family carers’ involvement in research, with no studies formally defining *impact* and only one study reporting formal measurement. Researchers and family carers appear to perceive impact differently: Researchers often focus on influences on study design, process, or outputs, whereas family carers highlight personal benefits such as empowerment, increased confidence, satisfaction and skill development. This divergence was also evident in our consultation. A similar picture emerges in other studies and reviews. Impact is not reported in a structured and regular manner [62, 65]; methods are not reported at all or are reported poorly [69]. When methods are reported, they are primarily based on qualitative data [69], and impact is occasionally evaluated solely from the researchers’ perspective [62, 63]. Similar aspects to those described in the included studies can be found elsewhere. For example, family carers feel empowered by their involvement in research [63], perceive high levels of self‐confidence and satisfaction [70] and develop new skills [64].

## 5 Limitations

The literature search for the scoping review was partially limited: we excluded grey literature and did not use forward citation tracking, which may have led to the omission of additional initiatives; however, focusing on peer‐reviewed studies ensured sufficient methodological transparency and comparability of reported involvement processes. Furthermore, not all the references were screened by two people independently of each other. The data extraction and analysis of the included studies were mostly carried out by one person only. The consultation involved a small number of family carers with limited age diversity and may not reflect the full range of caregiving experiences. Findings from this phase should therefore be interpreted as exploratory and illustrative.

## 6 Conclusion

Although we identified a wide range of recent research involving family carers of people with dementia as co‐researchers, our analysis showed that many studies provide limited or heterogeneous reporting, making it difficult to draw firm conclusions about the quality or diversity of involvement. Despite increased activity in recent years, family carers are most often engaged in the execution phase and in the role of co‐thinker; levels of involvement across other phases, such as planning or dissemination, remain less frequently reported. Many studies lack explicit frameworks or definitions for research involvement, and strategies for involvement are mentioned, when described, are often brief and poorly theoretically framed. Barriers and enablers to meaningful involvement are rarely assessed, and reported impacts are heterogeneous, with carers’ and researchers’ perspectives differing.

To advance dementia care research involving family carers, future studies should prioritise clear and structured involvement strategies, explicitly consider carers’ capacities and lived experiences, and report barriers, enablers, and impacts in a systematic and transparent manner. Structured reporting and use of established frameworks would improve comparability, while comprehensive and sustained involvement across all project phases is likely to enhance the relevance and accessibility for both family carers and the people they support.

## Author Contributions

**Franziska Anushi Jagoda:** conceptualisation, writing – original draft, methodology, writing – review and editing, investigation, data curation. **Julian Hirt:** methodology, writing – review and editing, investigation. **Claudia Mueller:** writing – review and editing, supervision. **Margareta Halek:** supervision, writing – review and editing.

## Ethics Statement

Ethical approval for the family carers' consultation of this scoping review was obtained from the Ethics Committee of the German Society of Nursing Science (Deutsche Gesellschaft für Pflegewissenschaft) on 7 September 2023 (Approval No. 23‐029). All participants provided informed consent prior to participation.

## Conflicts of Interest

The authors declare no conflicts of interest.

## Supporting information

Supporting File 1

Supporting File 2

Supporting File 3

Supporting File 4

## Data Availability

The data that support the findings of this study are available from the corresponding author upon reasonable request. The study protocol for this scoping review is publicly available on the Open Science Framework (OSF) https://osf.io/z26vu. Data generated from the consultation with family caregivers are not publicly available due to privacy and confidentiality considerations.
