# Supplementary material for: Family Carer Involvement in Dementia Care Research: A Scoping Review and Expert Consultation
Source: Health Expect. 2026 Jun 30;29(4):e70741. doi: 10.1111/hex.70741 (PMC13316458; doi:10.1111/hex.70741)
Supplement: Supplementary file 1 — Supporting File 1 [file HEX-29-e70741-s003.docx]

**APPENDIX**

**Consultation of family carers of people with dementia**

**Full Results**

The consultations took place in December 2024. As we were unable to find a joint meeting with four family carers, the group was split into two meetings, so that two consultations were held with two family carers each. The consultation lasted on average 49 minutes (consultation 1: 56 minutes; consultation 2: 42 minutes). There was one man and one woman in each of the two groups, and all but one of the family carers were former carers. The average age of the family carers was 63 years (range 60-66 years), their highest level of education was a university degree, and they were or had been the main family carer of the person with dementia providing care both within and outside the household. The people with dementia were mothers and one father.

All four family carers felt that it was important for researchers to consider strategies for involving family carers as co-researchers. It was important to them that participatory research would not be done spontaneously or on instinct, otherwise the strategies may not be well thought out. Researchers should consider and think about how to improve the accessibility of research projects for family carers. However, they themselves know how difficult it would be to involve family carers in research in the first place, so it is not 'very important'. The fact that 6 out of 40 studies explicitly reported on strategies was perceived by the family carers as very little. They did not understand why there were so few studies as it would still be difficult to involve family carers in research to this day. It is possible that researchers are not aware of this problem, although from a researcher's point of view it could be important to make strategies explicit so that other researchers can learn from them. It was also fundamental to the family carers to reflect on how a collaboration came about in the first place and at what level. Strategies for this should be made explicit. However, the family carers acknowledged that the lack of strategies could also be due to the publication logic.

All four family carers felt that it was very important for researchers to consider the level of involvement and therefore the different roles of family carers in a research project. For them, this would be a key aspect of participatory research. Transparency in this respect would also be fair to the co-researchers in research projects. It would also be important to be able to assess the contribution of co-researchers. Family carers should be clear from the outset about the effort they will have to put in as co-researchers, and this could be made clear on the basis of roles, as it would also be an internal conflict to care for someone and work on a research project at the same time. Researchers should decide together with the co-researchers in which role the family carer would like to participate, as this may change during the research project depending on the situation at home. Reflecting on his own situation, one former family carer pointed out that he would not have been able to participate in research for some time because of the caring situation at home. Family carers were not surprised by the division of roles in the included studies. As the responsibility and effort involved in the roles increases, the number of family carers would understandably decrease, as this would also require time and cognitive effort. Family carers felt that it could sometimes be difficult to facilitate a higher level of participation. However, they were pleasantly surprised that at least a quarter of the roles in the included studies were advisor roles. For the family carers who took part in the consultation, the role of a decision-maker would be unthinkable because they considered the effort involved to be too great, too demanding. It would require too much preparation. The family carers felt that it would certainly be time-consuming for researchers to find suitable formats for participation for each role, but for the family carers it would also be important to be taken seriously in their role.

Three of the four family carers felt that it was very important for researchers to deal with enablers in order for them to be co-researchers in the first place. They are so involved in the acute care situation that it is not easy for them to participate in research. Even if they wanted to, if the circumstances weren't right, they wouldn't be able to. The research context would always be secondary to them, so researchers should offer something to support them. Especially in the roles of decision-maker and partner, such facilitators would be very important, as family carers have a greater burden here. For family carers who work, the last two roles would be very difficult to implement, as they would have to familiarise themselves well with the project and may have to give up part of their job for the time. This would not be necessary for the co-thinker and advisor roles. Online formats, for example, would be a good alternative so that family carers don't have to leave home. These structures would need some lead time in a project to be designed and implemented. In the worst-case scenario, carers would also feel not taken seriously by the researchers if their situation at home was not sufficiently taken into account, as if they had not engaged sufficiently with the co-researchers to understand the situation. This would be more indicative of tokenistic inclusion. Family carers felt that anticipating facilitators was important but also difficult from the researcher's point of view because they would not be in the situation and therefore the situation at home would be more abstract and not so tangible for the researchers. However, these facilitators could only be made explicit in the exchange, so they would only partially develop during the process. One of the family carers thought that it was not so important to think about facilitators for the roles of co-thinker and advisor, as the effort for the relatives would not be particularly high and they would participate out of their own interest, so they could at least make things possible in these roles. The family carers felt that there were still some gaps in the area of participatory research with family carers, as the number of studies that would say something about facilitators is very small. The family carers felt that researchers should definitely think about facilitators depending on the level of involvement.

Three of the four family carers felt it was very important for researchers to think about barriers to participatory research. From a researcher's perspective, this may be even more important than looking at facilitators. One family carer thought that it was also important to talk about barriers at the end of the project, but more from the researcher's perspective than from the family carer's perspective. The family carers believed that dealing with barriers was just as important as dealing with facilitators. It would be important to find a common line. One of the family carers felt that it might not be so important to deal with barriers after a project because the project was over and there was no benefit for the family carers to talk about it at that point. The family carers were surprised that so few studies in this field provide information on both facilitators and barriers. After all, they are the main people who can provide insight and the difficult situation of family carers is well known. From the family carer's point of view, facilitators and barriers are a central issue, without which they could not be involved in an appropriate way. It may be that the lack of this discussion in the studies is due to the publication logic and therefore no reflection has taken place. It is also particularly important since family carers have no direct, individual benefit from participating, except perhaps the conversations they can have in the context of the research project. The family carers felt that a good study should also highlight the difficult aspects of the project and the strategies used to avoid these stumbling blocks. This is also important for other researchers. They should also learn from their mistakes in order to better involve family carers in the future.

In terms of impact, family carers felt that for the role of co-thinker and advisor it was not important how much impact their input had. They would share their thoughts and opinions and then it would not matter to them what happened. For the roles of partner and decision-maker, it would be important because the investment for the family carer would also be higher for these roles. As a family carer, you would also want your thoughts and opinions to have a strong impact. If the family carer's influence is low, the researchers might conclude that the approach was perhaps not really participatory. But the family carers would see this as a more important aspect of the research. That they could be accountable for what was done in the project. Family carers felt that the impact of their work was always related to the research task or idea. Although this is important, it is often more in the context of the researcher's perspective. In general, this is all a process of negotiation that goes hand in hand with role clarification.

The family carers felt that the researchers seemed to think that it was important to report on the impact. However, they would like more researchers to look at this and to be clear about the impact that family carers have had, as it is important to know whether family carers' views have been adequately taken into account. This is also part of the researchers' self-reflection. It would be important to ask oneself why the results are the way they are, and at the same time what added value the role of partner and decision-maker brings. Family carers were surprised that there were so few studies dealing with this issue of impact, as almost any study designed to be participatory should deal with it.
