# Supplementary material for: Family Carer Involvement in Dementia Care Research: A Scoping Review and Expert Consultation
Source: Health Expect. 2026 Jun 30;29(4):e70741. doi: 10.1111/hex.70741 (PMC13316458; doi:10.1111/hex.70741)
Supplement: Supplementary file 2 — Supporting File 2 [file HEX-29-e70741-s002.docx]

**APPENDIX**

**Data charting tool**

**Table 1: Citation details**

| **#Citation** | **First author(s) surname** | **Year of Publication** | **Journal** | **Title** | **Country of study conduct** | **Co-Author family carer** |
| --- | --- | --- | --- | --- | --- | --- |
|  |  |  |  |  |  |  |

**Table 2: Research characteristics**

| **#Citation** | **Aim** | **Design** |
| --- | --- | --- |
|  |  |  |

**Table 3: Involvement characteristics**

| **#Citation** | **Who was engaged?** | **Total number of people involved** | **Geographical location (from which family caregivers were recruited)** | **Research cycle phase (Shipee et al., 2015)** | **Framework/Definition of involvement** |
| --- | --- | --- | --- | --- | --- |
|  |  |  |  |  |  |

| **Strategies to include family carers** | **Types of involving activity** | **Training of family carers** | **Frequency of involvement** | **Roles adopted (Smits et al., 2020)** | **Type of compensation** |
| --- | --- | --- | --- | --- | --- |
|  |  |  |  |  |  |

**Table 4: Family caregiver’s reflection of involvement**

| **#Citation** | **Barriers** | **Enablers** | **Data collection method** | **Overall perception of the involvement** |
| --- | --- | --- | --- | --- |
|  |  |  |  |  |

**Table 5: Researchers’ reflection of family caregiver’s involvement**

| **#Citation** | **Barriers** | **Enablers** | **Data collection method** | **Overall perception of the involvement** |
| --- | --- | --- | --- | --- |
|  |  |  |  |  |

**Table 6: Impacts of involvement**

| **#Citation** | **Definition of Impact** | **Impact reported (researchers)** | **Measurement** | **Impact reported (family carers)** | **Measurement** |
| --- | --- | --- | --- | --- | --- |
|  |  |  |  |  |  |
