# Supplementary material for: Family Carer Involvement in Dementia Care Research: A Scoping Review and Expert Consultation
Source: Health Expect. 2026 Jun 30;29(4):e70741. doi: 10.1111/hex.70741 (PMC13316458; doi:10.1111/hex.70741)
Supplement: Supplementary file 3 — Supporting File 3 [file HEX-29-e70741-s004.docx]

APPENDIX

**Deviations from the published protocol Jagoda et al., (2024)**

**Corresponding Author:** Franziska Anushi Jagoda, Witten/Herdecke University, [franziska.jagoda@uni-wh.de](mailto:franziska.jagoda@uni-wh.de)

**Background:** This document outlines all deviations from the scoping review protocol titled Involvement of family caregivers in dementia care research: a scoping review protocol" (Jagoda, F.A., Hirt, J., Mueller, C. et al. Involvement of family caregivers in dementia care research: a scoping review protocol. Syst Rev 13, 277 (2024). <https://doi.org/10.1186/s13643-024-02696-w>), which was published on November 11, 2024.

1. **Overview of Deviations**

| **Protocol Section** | **Planned (Protocol)** | **Actual (Review)** | **Rationale for Deviation** |
| --- | --- | --- | --- |
| Data Extraction | Less detailed extraction items | data item *family carer co-authorship* was added | to identify studies that partner with family carers or self-help groups |
|  |  | data item *strategies to include family carers* was added | to be able to answer the research questions for the scoping review better and more directly |
|  |  | data item *roles adopted* was added | to be able to answer the research questions for the scoping review better and more directly; Framework on roles provides the possibility of a frame of reference that can be used in the further course of the study |
|  |  | data item *method* was added | to be able to answer the question of how impact or reflections were recorded in the studies |

1. **Additional Deviations**

n.a.

1. **Impact on Scoping Review**

The deviations did not affect the overall objectives or conclusions of the scoping review. Including more data charting items increased comprehensiveness.

1. **Declaration**

All authors have reviewed and approved the deviations. The rationale for each change has been documented transparently.
