# Supplementary material for: Family Carer Involvement in Dementia Care Research: A Scoping Review and Expert Consultation
Source: Health Expect. 2026 Jun 30;29(4):e70741. doi: 10.1111/hex.70741 (PMC13316458; doi:10.1111/hex.70741)
Supplement: Supplementary file 4 — Supporting File 4 [file HEX-29-e70741-s001.docx]

**APPENDIX**

**Full database-specific search strategies**

| database | Search string |
| --- | --- |
| MEDLINE via PubMed | (famil*[TIAB] OR spouse*[TIAB] OR relative*[TIAB] OR informal[TIAB] OR household*[TIAB] OR dyad*[TIAB] OR caregiv*[TIAB] OR "care giver"[TIAB] OR carer*[TIAB] OR Family[MeSH] OR Caregivers[MeSH]) AND (dement*[TITLE] OR Alzheimer*[TITLE] OR dementia[MeSH]) AND (involv*[TITLE] OR PPI[TITLE] OR engag*[TITLE] OR participat*[TITLE] OR "co-produc*"[TITLE] OR "co-design*"[TITLE] OR collaborat*[TITLE] OR cooperat*[TITLE] OR "emancipatory"[TITLE] OR "user-led"[TITLE] OR "action research"[TITLE] OR "advisory group*"[TITLE] OR consult*[TITLE] OR panel*[TITLE] OR partner*[TITLE] OR "experts by experience"[TITLE] OR "citizen science"[TITLE] OR Community-Based Participatory Research[MeSH] OR Patient Participation[MeSH] OR Citizen Science[MeSH]) |
| CINAHL | ((TI famil* OR AB famil*) OR (TI spouse* OR AB spouse*) OR (TI relative* OR AB relative*) OR (TI informal OR AB informal) OR (TI household* OR AB household*) OR (TI dyad* OR AB dyad*) OR (TI caregiv* OR AB caregiv*) OR (TI "care giver" OR AB "care giver") OR (TI carer* OR AB carer*) OR (MH Family+) OR (MH Caregivers+)) AND ((TI dement*) OR (TI Alzheimer*) OR (MH dementia+)) AND ((TI involv*) OR (TI PPI) OR (TI engag*) OR (TI participat*) OR (TI co-produc*) OR (TI co-design*) OR (TI collaborat*) OR (TI cooperat*) OR (TI emancipatory) OR (TI user-led) OR (TI "action research") OR (TI "advisory group*") OR (TI consult*) OR (TI panel*) OR (TI partner*) OR (TI "experts by experience") OR (TI "citizen science") OR (TI "Community-Based Participatory Research") OR (TI "Patient Participation") OR (MH "Action Research+") OR (MH "Citizen Science+") OR (MH "Consumer Participation+")) |
| Scopus (Elsevier) | (TITLE-ABS-KEY(famil*) OR TITLE-ABS-KEY(spouse*) OR TITLE-ABS-KEY (relative*) OR TITLE-ABS-KEY (informal) OR TITLE-ABS-KEY (household*) OR TITLE-ABS-KEY (dyad*) OR TITLE-ABS-KEY (caregiv*) OR TITLE-ABS-KEY ("care giver") OR TITLE-ABS-KEY (carer*)) AND (TITLE(dement*) OR TITLE(Alzheimer*)) AND (TITLE(involv*) OR TITLE(PPI) OR TITLE(engag*) OR TITLE(participat*) OR TITLE(co-produc*) OR TITLE(co-design*) OR TITLE(collaborat*) OR TITLE(cooperat*) OR TITLE(emancipatory) OR TITLE(user-led) OR TITLE("action research") OR TITLE("advisory group*") OR TITLE(consult*) OR TITLE(panel*) OR TITLE(partner*) OR TITLE("experts by experience") OR TITLE("citizen science")) |
| PsycInfo (EBSCO) | ((TI famil* OR AB famil*) OR (TI spouse* OR AB spouse*) OR (TI relative* OR AB relative*) OR (TI informal OR AB informal) OR (TI household* OR AB household*) OR (TI dyad* OR AB dyad*) OR (TI caregiv* OR AB caregiv*) OR (TI "care giver" OR AB "care giver") OR (TI carer* OR AB carer*) OR (DE Family) OR (DE Caregivers)) AND ((TI dement*) OR (TI Alzheimer*) OR (DE dementia) OR (DE "Alzheimer's Disease")) AND ((TI involv*) OR (TI PPI) OR (TI engag*) OR (TI participat*) OR (TI co-produc*) OR (TI co-design*) OR (TI collaborat*) OR (TI cooperat*) OR (TI emancipatory) OR (TI user-led) OR (TI "action Research") OR (TI "advisory group*") OR (TI consult*) OR (TI panel*) OR (TI partner*) OR (TI "experts by experience") OR (TI "citizen science") OR (DE "Action Research")) |
